# Serological Responses after a Fourth Dose of SARS-CoV-2 Vaccine in Solid Organ Transplant Recipients: A Systematic Review and Meta-Analysis

**DOI:** 10.3390/vaccines11071130

**Published:** 2023-06-21

**Authors:** Yameng Mu, Hongxiao Wu, Zhouling Jiang, Kehang Liu, Xiaoyu Xue, Wei Zhang, Zhihai Chen

**Affiliations:** Department of Infectious Disease, Beijing Ditan Hospital, Capital Medical University, Beijing 100102, China; muyameng1204@163.com (Y.M.);

**Keywords:** COVID-19, SARS-CoV-2, vaccine, the fourth dose, solid organ transplant recipients

## Abstract

The humoral immune response and safety of the fourth dose of the coronavirus disease 2019 (COVID-19) vaccine in solid organ transplant (SOT) recipients need to be fully elucidated. We conducted a systematic review and meta-analysis to assess the efficacy and safety associated with this additional dose of the COVID-19 vaccine in the SOT recipients. A comprehensive search was conducted to identify studies on SOT patients without prior natural SARS-CoV-2 infection who received the fourth dose of the COVID-19 vaccine. Serological antibody responses following vaccination were synthesized by a meta-analysis of proportions. The proportions for each outcome were integrated by using a random-effects model. Approximately 56–92% of the SOT patients developed a humoral immune response, and the pooled seroprevalence rate was 75% (95% confidence interval [CI], 62–82%) after administering the third vaccine dose. Following the fourth dose of vaccination, approximately 76–95% of the patients developed a humoral immune response. The pooled seroprevalence rate after the fourth dose was 85% (95% CI, 79–91%). Of the patients who initially tested seronegative after the second dose, approximately 22–76% of patients subsequently became seropositive after the third dose. The pooled seroconversion rate for the third dose was 47% (95% CI, 31–64%). Among the patients who were seronegative after the third dose, approximately 25–76% turned seropositive after the fourth dose. The pooled seroconversion rate after the fourth dose was 51% (95% CI, 40–63%). Safety data were reported in three studies, demonstrating that adverse effects following the fourth dose were generally mild, and patients with these adverse effects did not require hospitalization. No transplant rejection or serious adverse events were observed. A fourth dose of the COVID-19 vaccine in SOT recipients was associated with an improved humoral immune response, and the vaccine was considered relatively safe.

## 1. Introduction

Solid organ transplant (SOT) recipients are immunocompromised because of the long-term use of immunosuppressive drugs. SOT recipients with the coronavirus disease 2019 (COVID-19) infection face increased risks of severe disease, mortality, and prolonged viral shedding [1,2]. Vaccination can reduce virus transmission, prevent infection, and improve clinical outcomes in the general population [3,4]. Compared with the general population, SOT recipients experience decreased immunogenicity after COVID-19 vaccination and a significant proportion of patients remain seronegative [5,6].

The seroprevalence and seroconversion of SOT patients after vaccination have currently attracted increasing attention. Lee et al. [7] found that after one vaccine dose, transplant recipients were 16 times less likely to seroconvert than immunocompetent controls. Moreover, the likelihood of seroconversion in transplant recipients who received the second dose remained minimal, with only one in three patients achieving seroconversion. A meta-analysis revealed the weighted mean of the positive humoral response after the administration of the mRNA COVID-19 vaccine was 10.4% for the first dose, 44.9% for the second dose, and 63.1% for the third dose [8]. In addition, another study revealed that SOT vaccinees exhibited humoral immune response rates of 9.5%, 43.6%, and 55.1% after the first, second, and third doses of the COVID-19 vaccine, respectively [9]. However, the protective efficacy of the vaccine for SOT patients’ needs to be further improved.

To date, the efficacy and safety of a fourth dose of the COVID-19 vaccine in SOT patients have not been systematically evaluated. This is despite studies exhibiting increased seropositivity rates in SOT recipients who received higher vaccine doses [10]. Because of reduced immunogenicity, we remain concerned that the protection provided by the fourth vaccine dose to SOT recipients is inadequate, and these patients are at risk for breakthrough infections or adverse clinical outcomes. Therefore, we conducted this systematic review and meta-analysis to summarize the current evidence on the response to the fourth COVID-19 vaccine dose.

## 2. Methods

This systematic review is reported according to the Preferred Reporting Items for Systematic Reviews and Meta-Analyses guidelines [11]. Before the study commencement, the protocol was registered with PROSPERO, a prospective international register of systematic reviews (CRD42023381608).

### 2.1. Search Strategy

According to the study protocol, articles published between 1 December 2020 and 10 February 2023 were thoroughly searched across multiple databases, including PubMed, Embase, and the Cochrane Central Register of Controlled Trials. The search used a range of keywords including (“COVID-19” or “COVID 19” or “COVID2019” or “COVID 2019” or “COVID-2019” or “Novel Coronavirus” or “new coronavirus” or “novel corona virus” or “new corona virus” or “SARS-CoV2” or “SARS-CoV-2” or “SARS CoV 2” or “SARSCoV2” or “2019-nCoV” or “2019nCoV” or “2019 coronavirus” or “2019 corona virus” or “Coronavirus Disease 2019” or “Disease 2019, Coronavirus” or “Coronavirus Disease-19” or “Coronavirus Disease 19” or “severe acute respiratory syndrome coronavirus 2” or “SARS Coronavirus 2” or “sars-coronavirus-2” or “Corona virus Disease 2019”) AND (“Vaccination” or “Vaccines” or “vaccine” or “vaccination” or “mRNA-1273” or “BNT162b2” or CoronaVac”) AND (“Transplants” or “Transplantation” or “Organ Transplantation”). No restrictions were placed on the language of publication.

### 2.2. Study Selection

First, two researchers (Y.M. and Z.J.) independently screened the titles and abstracts of each article. The full articles were reviewed only when the inclusion criteria were met or any uncertainty was encountered. A third researcher (K.L.) resolved discrepancies through consensus. Only studies involving human participants and any follow-up times and time points were included. 

We conducted a meta-analysis of several observational studies that met the following criteria: SOT recipients who received a fourth dose of any brand and type of the COVID-19 vaccine and at least reported seroconversion or serologic titers after COVID-19 vaccination.

Studies involving SOT recipients with prior natural SARS-CoV-2 infection or those vaccinated before transplantation were excluded. When data were unavailable for a particular study, the corresponding author was contacted for information. We excluded studies for which data were unavailable during the meta-analysis.

### 2.3. Data Extraction

The two researchers (Y.M. and Z.J.) extracted the data using a standardized predetermined data extraction sheet. At the end of data extraction, the two researchers reviewed all key data and assessed article quality. The data included the name of the first author, publication year, study location, study design, sample size, inclusion and exclusion criteria, age, sex, organ transplant type, transplant time, immunosuppressive drug regimen, vaccine type, number of participants receiving each vaccine type, the time interval between the third and fourth doses, assay type, serological response, antibody titer, measurement method, the time interval between sample collection, and adverse effects (AEs).

### 2.4. Outcomes

The primary study outcomes were serological responses after the fourth vaccine dose and comparison with the third dose. To assess the serological response, key parameters including seroprevalence rates, seroconversion rates, and mean or median serological antibody titers were analyzed. The seroprevalence rate is the ratio of seropositive patients after vaccination to vaccinated patients. The seroconversion rate refers to the ratio of patients who were first seronegative but tested seropositive after revaccination to those who were revaccinated. For each study, serological positivity was determined based on the manufacturer’s thresholds. Because antibody types and units of measurement differed between studies, we could not perform quantitative analyses. The antibody titers after the third and fourth vaccine doses were summarized in a descriptive form. The secondary outcome was the incidence of adverse reactions after the fourth vaccine dose.

### 2.5. Data Analysis

All data were analyzed using Stata version 17.0. Pooled proportions and corresponding 95% confidence intervals (CIs) for the primary outcomes of interest were estimated using random-effects models. Statistical heterogeneity of the results of the included studies was assessed using the χ^2^ test and I^2^ statistic. Heterogeneity was considered significant when the *p*-value of the χ^2^ test was <0.10 or when the I^2^ statistic was ≥50%. Sensitivity analysis was performed by removing a study to confirm whether the results were reliable. Given the variation in the antibody types and units of measurement across the studies, statistic pooling of antibody titers was anticipated to be inappropriate. Therefore, antibody titers were evaluated using an integrated approach without meta-analysis. Because this was a meta-analysis of proportions, publication bias was not assessed. All analyses were performed using the “metan” and “metainf” commands on Stata version 17.0. *p* < 0.05 was considered statistically significant.

### 2.6. Assessment of Study Quality

The quality of the included studies was assessed according to the Methodological Index for Non-Randomized Studies criteria. The global ideal scores for non-comparative and comparative studies are 16 and 24, respectively. Among the nine studies, three studies were scored as high quality [12,13,14], three studies as moderate quality [15,16,17], and the remaining three studies as low quality [18,19,20] (Table 1). The risk of bias was thus acceptable.

## 3. Results

**Figure 1:**
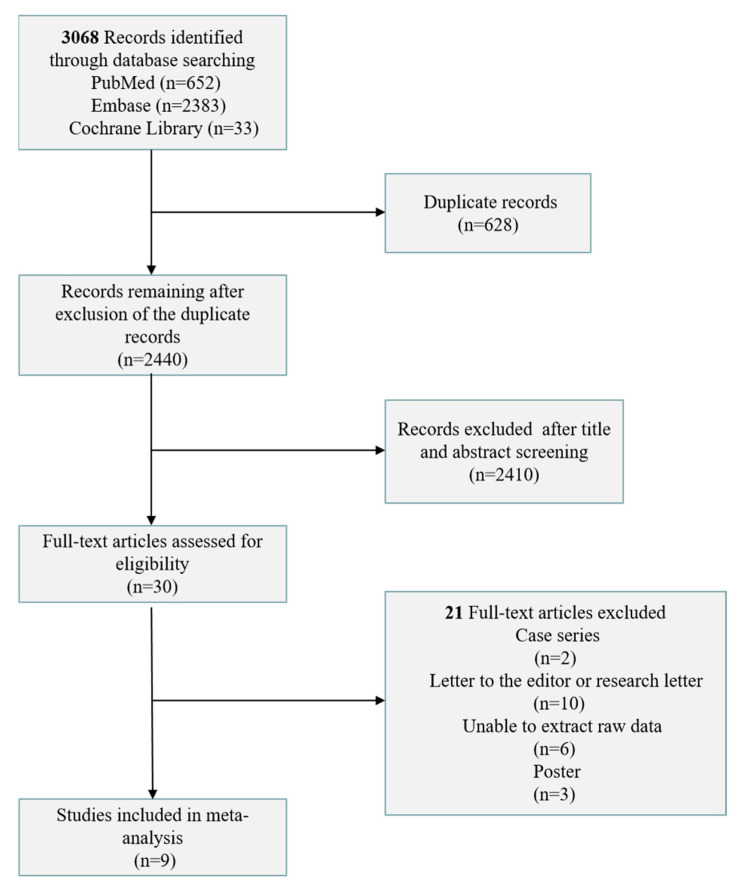
Flow diagram depicting the study selection process.

A total of 3068 articles from the database were comprehensibly reviewed. After removing duplicate articles and critically evaluating the titles and abstracts, 21 articles were selected for a thorough evaluation. Overall, nine studies were included in the meta-analysis after rigorous full-text screening (Figure 1).

### 3.1. Characteristics of the Included Studies

Table 2 presents the details of the included studies. This meta-analysis involved 1917 transplant recipients who received a third vaccine dose and 1307 transplant recipients who received a fourth vaccine dose. All nine studies were published in 2022. Of them, five studies included prospective single-arm cohorts [12,15,18,19,20]. Of the remaining four studies, three studies included retrospective single-arm cohorts [13,16,17], and one was a prospective cohort study that compared liver transplant recipients with healthy controls [14]. Kidney transplantation was the predominant organ transplantation among the included studies. Across all studies, 44–70% of patients were men. The median or mean age of the patients was 55–65 years. In most studies, patients received mRNA vaccines, except for three studies in which the adenoviral vector vaccine ChAdOx1-S or Ad26.COV2.S was administered [15,17,18]. The time interval between the administration of the third and fourth vaccine doses was 55–201 days. 

### 3.2. Primary Outcome Measurement

Key measurements of the serological response included the seroprevalence rate, seroconversion rate, and antibody titer levels. A study consistently documented the serological response from the first to the fourth vaccine dose [16]. Another study reported humoral immune responses following the administration of the second, third, and fourth vaccine doses [13]. Other articles documented antibody immune responses following the administration of the third and fourth vaccine doses. The time interval between the third vaccine dose and testing varied from 14 to 173 days. A serological response is usually tested 16–32 days after the fourth vaccine dose is administered. In one study, chemiluminescence analysis [18] was used for serological response measurements, while the enzyme-linked immunosorbent assay was used in other studies. 

### 3.3. Seroprevalence Rate in SOT Recipients 

**Figure 2:**
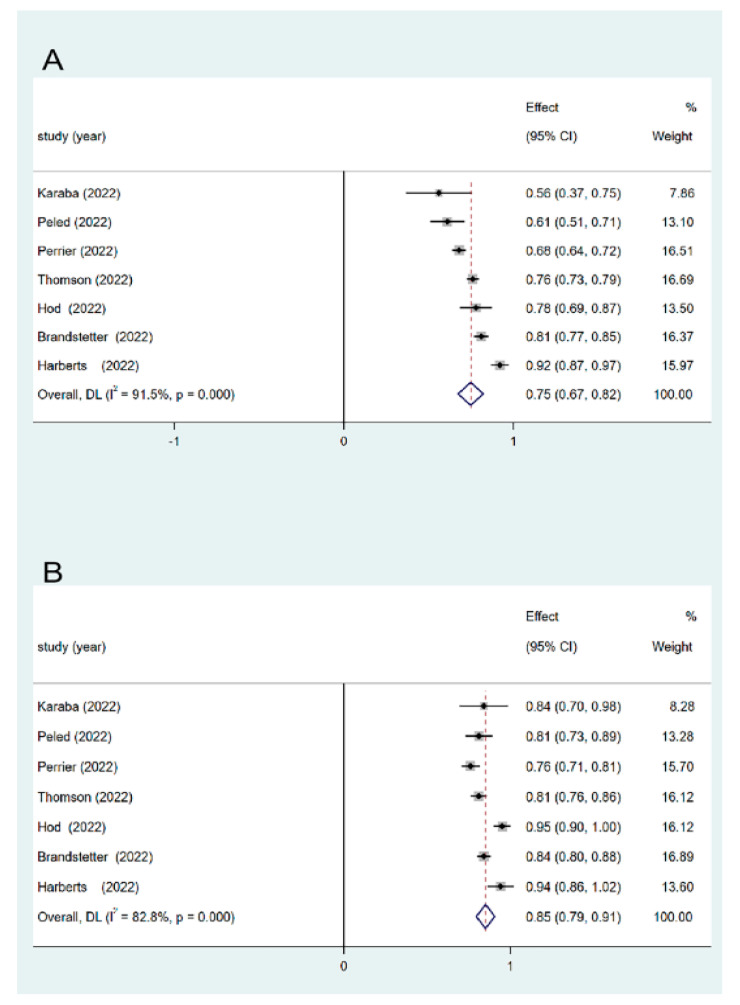
Meta-analysis of the seroprevalence after third and fourth doses of COVID-19 vaccine in SOT recipients and the comparison of seroprevalence. (**A**) Seroprevalence rate after the third vaccine dose in SOT (75%). (**B**) Seroprevalence rate after the fourth vaccine dose in SOT (85%) [12,13,14,15,16,18,19]. SOT, solid organ transplant; CI, confidence interval; DL, DerSimonian-Laird.

**Figure 3:**
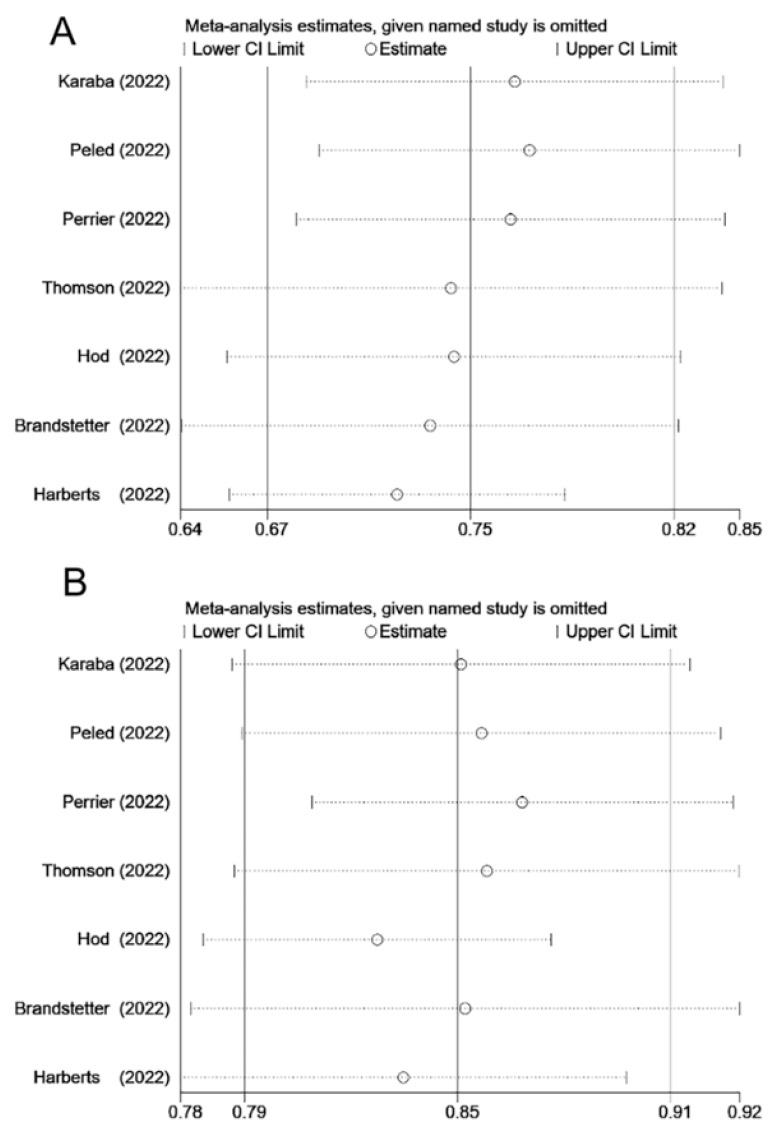
Sensitivity analysis of the seroprevalence rate after the third COVID-19 vaccine dose (**A**) and the fourth COVID-19 vaccine dose (**B**) among SOT recipients [12,13,14,15,16,18,19]. SOT, solid organ transplant; CI, confidence interval.

In seven studies, seroprevalence rates were assessed in the SOT recipients after the third and fourth vaccine doses were administered. Following the administration of the third vaccine dose, approximately 56–92% of the patients developed a humoral immune response (Table 3). The pooled seroprevalence rate for the third vaccine dose was 75% (95% CI, 62–82%) (Figure 2). Considerable heterogeneity was observed (I^2^ = 91.5%, *p* < 0.001). Sensitivity analysis exhibited no significant changes (Figure 3). Approximately 76–95% of the patients developed a serological response after the fourth dose of vaccination. The pooled seroprevalence rate after the fourth vaccine dose was 85% (95% CI, 79–91%) (Figure 2). Considerable heterogeneity was observed (I^2^ = 82.8%, *p* < 0.001). No significant change was observed in the sensitivity analysis results (Figure 3). 

### 3.4. Seroconversion Rate in SOT Recipients 

**Figure 4:**
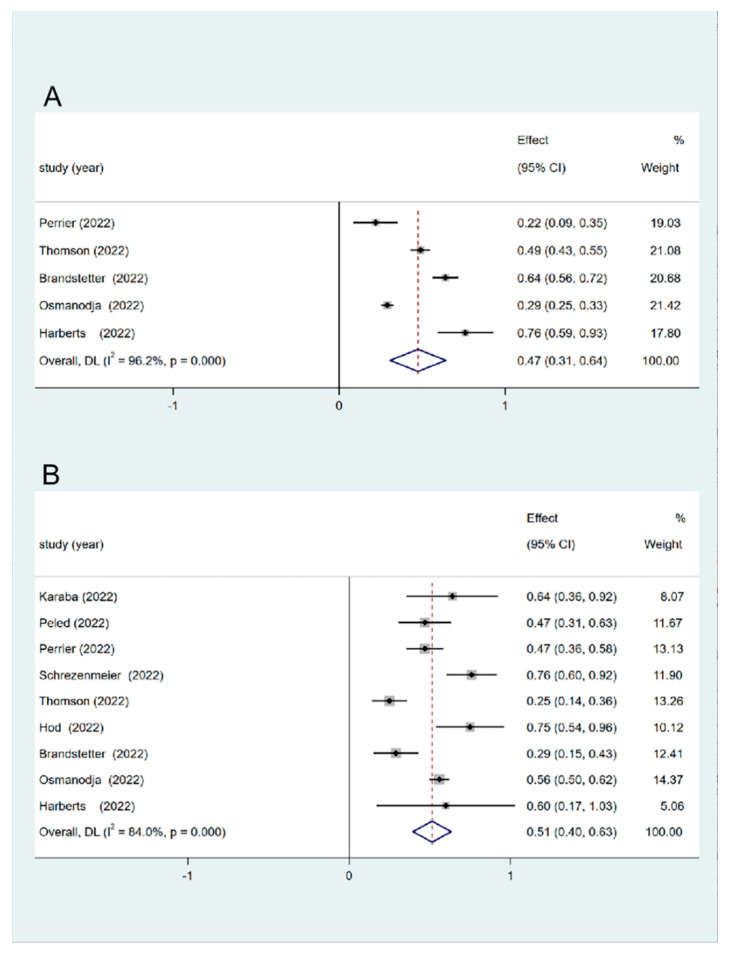
Meta-analysis of the seroconversion rate after the third and fourth doses of the COVID-19 vaccine in SOT recipients and the comparison of seroconversion. (**A**) Seroconversion rate after the third vaccine dose in SOT (47%). (**B**) Seroconversion rate after the fourth vaccine dose in SOT (51%) [12,13,14,15,16,17,18,19,20]. SOT, solid organ transplant; CI, confidence interval; DL, DerSimonian-Laird.

**Figure 5:**
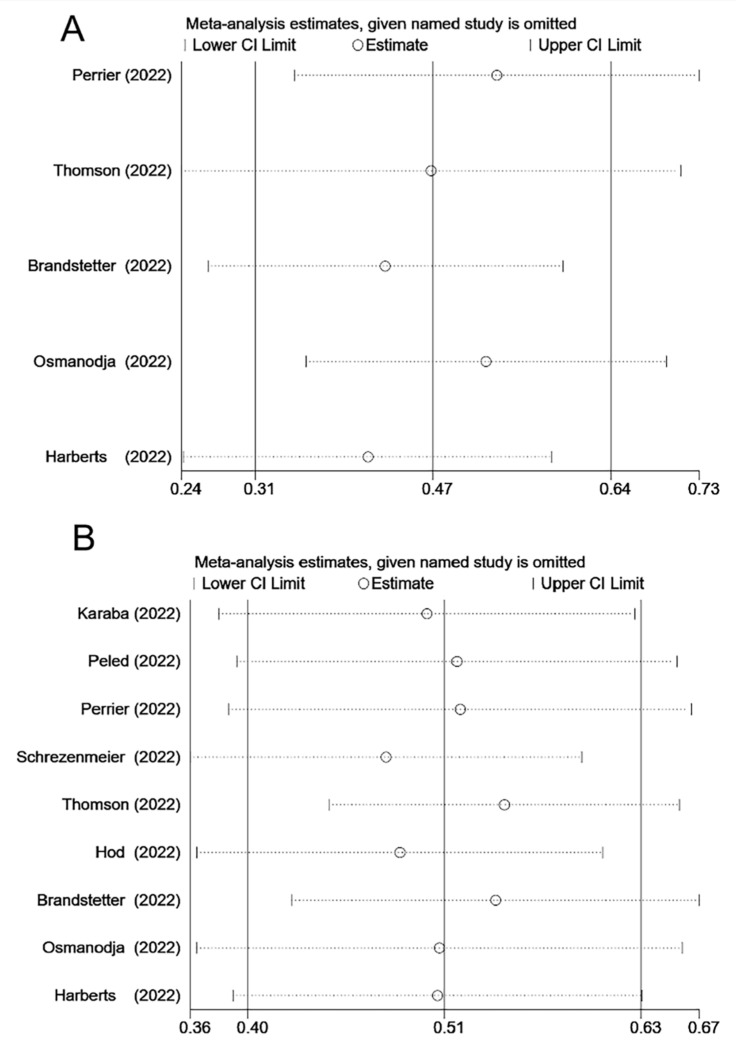
Sensitivity analysis of seroconversion rate after the third COVID-19 vaccine dose (**A**) and the fourth COVID-19 vaccine dose (**B**) among SOT recipients [12,13,14,15,16,17,18,19,20]. SOT, solid organ transplant; CI, confidence interval.

In five studies, seroconversion rates were reported after the third dose of vaccination. In all nine studies, the seroconversion rate was assessed after the fourth dose. Among these studies, two studies reported higher seroconversion rates for the fourth dose than for the third dose [16,17]. However, in the other three studies, the seroconversion rate for the fourth dose was lower than that for the third dose [13,14,15]. Approximately 22–76% of the patients who initially tested seronegative after the second vaccine dose and subsequently turned seropositive after the third dose (Table 3). The pooled seroconversion rate for the third dose was 47% (95% CI, 31–64%) (Figure 4) with considerable heterogeneity (I^2^ = 96.2%, *p* < 0.001). The sensitivity analysis exhibited no significant changes (Figure 5). Among the patients who remained negative after the third dose of vaccination, approximately 25–76% became seropositive after the fourth dose. The pooled seroconversion rate after the fourth dose was 51% (95% CI, 40–63%) (Figure 4) with considerable heterogeneity (I^2^ = 84.0%, *p* < 0.001). The sensitivity analysis revealed no significant changes (Figure 5).

### 3.5. Antibody Titers in SOT Recipients

In this systematic review and meta-analysis, the included studies exhibited variations in both the detection type and the unit of measurement of SARS-CoV-2 antibodies. As shown in Table 3, two studies reported anti-Spike IgG antibodies [15,18], while five studies detected anti-receptor-binding domain antibodies [12,13,14,18,19]. Moreover, one study measured the neutralizing antibody through the SARS-CoV-2 pseudovirus neutralization assay [12]. The unit of measurement varied across the studies, with three studies using BAU/mL [13,15,18], two studies using AU/mL [14,19], and one study not specifying a unit of measurement [12]. Therefore, a meta-analysis of antibody titers was excluded. Table 3 depicts the antibody titer levels for each study. Three studies reported higher antibody titers in the SOT patients after the fourth dose of vaccination than the third dose of vaccination [15,18,19]. By contrast, the other two studies reported conflicting results [13,14].

### 3.6. Factors Associated with a Humoral Immune Response after Vaccination in SOT Patients

In total, two studies reported factors associated with improved humoral immune responses after both the third and fourth doses of vaccination (Table 4). Among the host characteristics, increased age and diabetes history were associated with lower seropositivity after the third vaccine dose was administered. Low positive anti-SARS-CoV-2-S-protein IgG levels before vaccination, higher body mass index, higher estimated glomerular filtration rate (eGFR), and higher hemoglobin levels were associated with an improved serologic response after the third dose of vaccination. Increasing age was associated with a lower humoral immune response after the fourth dose of vaccination. Low positive anti-SARS-CoV-2-S-protein IgG levels before vaccination were associated with an improved humoral response after the fourth dose of vaccination. Regarding transplant characteristics, older age at transplantation was associated with an improved humoral immune response after the vaccination. Vaccination within the first year after transplant was an independent risk factor for non-seroconversion. Regarding immunosuppressive drugs, calcineurin inhibitor (CNI) monotherapy was associated with an improved humoral immune response. However, belatacept treatment and higher mycophenolate (MPA) doses were associated with decreased serologic responses after the third and fourth doses.

### 3.7. Safety

Three studies reported AEs following vaccination (Table 3). Harberts et al. [14] reported local and systemic AEs in follow-up transplant patients who received the third or fourth vaccination. They developed mild AEs, such as pain or swelling at the injection site, fatigue, headache, vomiting, muscle pain, joint pain, and diarrhea, that did not require hospitalization. One study reported AEs in approximately 82.4% of the recipients after the fourth dose of the BNT162b2 vaccine was administered. Local and systemic AEs were reported in 75.7% and 37.8% of the cohort, respectively. Injection site pain was the most common local adverse event and was reported by approximately 75.7% of these patients. Fatigue was the main systemic AE reported by 27% of the patients. No cases of rejection or allergic reactions were noted [12]. Furthermore, one study documented a patient diagnosed with biopsy-proven acute rejection, classified as Banff Ia, 2 weeks after the third vaccination. This may be associated with undergoing primary kidney transplantation approximately 1 year before the third vaccination. No transplant rejection was observed after the fourth vaccination [13].

## 4. Discussion

SOT recipients with COVID-19 infection are prone to an elevated risk of severe disease and mortality [21,22]. Unfortunately, SOT recipients are often excluded from phase III clinical trials conducted before vaccine marketing. Consequently, the efficacy and safety of COVID-19 vaccination in the SOT population is of great concern. Studies have explored serum immune responses after the first, second, and third doses of the COVID-19 vaccine were administered in SOT patients [23,24] and have demonstrated that COVID-19 vaccination is relatively safe in SOT recipients. However, the immune response was inadequate and failed to provide sufficient protection. The incidence of breakthrough infection was higher among fully vaccinated SOT recipients than among the general population [5]. The second dose of the vaccine was effective in SOT patients [25,26]. Meanwhile, a third vaccine dose improved the serological response in the transplant recipients compared with the second dose [27]. However, this response was considered suboptimal because a significant proportion of patients failed to produce detectable antibodies after the third dose vaccination, thereby increasing the hope for an additional benefit of a fourth dose in the SOT population. Therefore, the current focus is on identifying the potential advantages of a fourth dose of vaccination in SOT patients. However, little information is available on seroprevalence and seroconversion rates in SOT patients who receive the fourth dose of the COVID-19 vaccine. Systematic reviews or meta-analyses on the efficacy and safety of a fourth vaccine dose in SOT patients are lacking. This is the first study to systematically investigate the efficacy and safety and highlight the importance of a fourth vaccine dose in SOT patients.

Indicators such as the seroprevalence rate, seroconversion rate, and antibody titers were used to evaluate the effectiveness of COVID-19 vaccines. In our study, the fourth vaccine dose significantly improved both seroprevalence and seroconversion rates in the SOT recipients. The fourth dose of vaccination is associated with increased immunogenicity and protection. In the present systematic review and meta-analysis of nine studies, the seroprevalence rate of the COVID-19 vaccine among the SOT recipients was 75% (95% CI, 0.62–0.82) after the third dose and 85% (95% CI, 0.79–0.91) after the fourth dose. Seroconversion rates were 47% (95% CI, 0.31–0.64) and 51% (95% CI, 0.40–0.63) after the third and fourth doses, respectively. Humoral immune responses improved in the SOT recipients after the fourth dose. These findings suggest that a second booster vaccination should be considered in SOT patients to improve their serum immunity levels.

Our further analysis revealed several risk factors associated with low seroconversion rates in SOT patients after the third and fourth COVID-19 vaccination. Different transplant types affect the seroconversion rate of patients after vaccination, and liver transplant patients had a stronger humoral response after the second, third, and fourth doses of the SARS-CoV-2 vaccine (OR = 5.3, 3.7, and 6.6, respectively, compared with other organ transplant recipients, *p* < 0.001). In addition, low positive anti-SARS-CoV-2-S-protein IgG levels before vaccination, higher eGFR, higher hemoglobin levels, and CNI monotherapy were associated with improved serologic responses after SARS-CoV-2 vaccination in the SOT patients. However, old age, diabetes, administration of belatacept, and high MPA doses were associated with reduced serological responses after the third and fourth vaccination in the SOT patients. One study exhibited a weak antibody response to three mRNA vaccine doses in belatacept-treated kidney transplant recipients [28]. Lower seroconversion with these agents (belatacept and MPA) could be caused by direct suppression of the B-lymphocyte function or suppression of T-lymphocyte-dependent B-lymphocyte activation [29,30]. Studies investigating the serological response of different immunosuppressive and immunosuppressive regimens in SOT patients after the administration of four doses of the SARS-CoV-2 vaccine are warranted.

Three studies reported AEs after the fourth dose of vaccination. These AEs were generally mild and did not need hospitalization. Moreover, no transplant rejection or serious adverse events were observed. Injection site pain was the most common local AE, while fatigue was the most common systemic AE. The safety profile of the fourth vaccine dose in the transplant recipients was consistent with that of the first, second, and third doses. In a real-world study including 19,586 general participants, approximately 64.9% of people experienced AEs after the first dose of BNT162b2 or mRNA-1273 vaccination. AEs were reported in approximately 80.3% of patients after they received the second dose of the COVID-19 vaccine. The most common adverse events were fatigue, muscle pain, headache, chills, injection site redness, joint pain, and fever. Other AEs included nausea, vomiting, diarrhea, dizziness, brain fog, swollen lymph nodes, and injection site pain or soreness. Serious adverse events were rare [31]. Massa et al. [32] reported AEs in 73%, 68%, and 68% of the SOT patients after the first, second, and third vaccine injections, respectively. Injection site pain was the most common AE. At follow-up, one participant developed new donor-specific antibodies, and this patient retrospectively confessed to not adhering to immunosuppressive drugs during vaccination.

This study has several limitations. First, studies investigating the serological response to the fourth dose of vaccine in SOT recipients are limited. Second, the studies included in this meta-analysis were all observational; six of them were prospective cohort studies, three were retrospective cohort studies, and none were randomized controlled trials. Last, a considerable degree of heterogeneity was observed between the studies.

## Figures and Tables

**Table 1:** Assessments of risks of bias of eligible studies in accordance with the revised and validated version of MINORS.

	Clearly Stated Aim	Inclusion of Consecutive Patients	Prospective Data Collection	Endpoints Appropriate to Study Aim	Unbiased Assessment of Study Endpoint	Follow-Up Period Appropriate To Study Aim	<5% Loss to Follow-Up	Prospective Calculation of Study Size	Adequate Control Group	Contemporary Groups	Baseline Equivalence of Groups	Adequate Statistical Analyses	Total Score	Quality
Karaba et al. [18]	2	1	2	1	1	1	2	0	0	0	0	0	10	low
Peled et al. [19]	2	1	2	2	1	1	2	1	0	0	0	0	12	low
Perrier et al. [16]	2	2	2	2	2	1	1	1	1	1	1	2	18	moderate
Schrezenmeier et al. [20]	2	1	2	2	1	2	2	0	0	0	0	0	12	low
Thomson et al. [15]	2	1	2	2	2	1	1	0	1	2	2	2	18	moderate
Hod et al. [12]	2	2	2	2	1	1	2	1	2	2	2	2	21	high
Brandstetter et al. [13]	2	1	2	2	2	1	1	1	2	2	2	2	20	high
Osmanodja et al. [17]	2	2	2	2	1	1	1	1	0	0	0	2	14	moderate
Harberts et al. [14]	2	2	2	2	1	2	1	0	2	2	2	2	20	high

The items are scored 0 (not reported), 1 (reported but inadequate) or 2 (reported and adequate). The global ideal score is 24 for comparative studies. The corresponding scores are 0–6, very low quality; 7–12, low quality; 13–18, moderate quality; and 19–24, high quality.

**Table 2:** Baseline characteristics of the enrolled studies and the study participants.

Study	Country	Population	Sample Size	Male (%)	Age (y), Mean ± SD/ Median (IQR/Range)	Time Post-Transplant (y), Mean ± SD/ Median (IQR/Range)	Type of Vaccine	Immunogenicity Detection Method	Time Interval between 3–4 Dose (d), Mean ± SD/ Median (IQR)	Immunotherapy Drugs
Karaba et al. [18]	USA	KT LIT HT LUT PT	25	11 (44%)	59 (45–66)	4.3 (2.7–8.8)	BNT162b2 mRNA-1273 Ad26.COV2.S (D3)	CLIA	93 (28–134)	Prednisone (72%) CNI (96%) MMF (84%) mTOR inhibitor (8%) Belatacept (4%)
Peled et al. [19]	USA	HT	90	62 (68.9%)	57.2 ± 13.8	6.5 (3.5–14.1)	BNT162b2	ELISA	173.4 ± 4.2	CNI + MPA + prednisone (54.4%) CNI + MPA (21.1%) CNI + everolimus + prednisone (15.7%) MPA + everolimus + prednisone (2.2%) Everolimus + CNI (3.3%) Everolimus + MPA (1.1%) CNI + prednisone (2.2%)
Perrier et al. [16]	France	KT LIT HT LUT	Total: 825 D3: 257 D4: 523	550 (66.7%)	61.2 (50.9–69.3)	6.7 (3.3–11.9)	BNT162b2	ELISA	201 (173–221)	NA
Schrezenmeier et al. [20]	Germany	KT	29	17 (58.6%)	59.8 ± 14.8	9.9 ± 5.9	BNT162b2	ELISA	59.1 ± 12.6	Tac + MPA (13.8%) CS + Tac + MPA (51.7%) CS + CyA + MPA (20.7%) CyA + MPA (3.4%) Belatacept + Aza ± CS (3.4%) Belatacept + MPA ± CS (6.8%) Belatacept + MPA (3.4%)
Thomson et al. [15]	UK	KT	D3: 586 D4: 239	D3: 384 (65.5%) D4: 149 (62.3%)	NA	NA	BNT162b2 mRNA-1273 ChAdOx1-S	ELISA	NA	CNI D3: (46.6%); D4: (40.6%) CNI + MMF/Aza D3: (26.6%); D4: (31.4%) CNI + MMF + prednisone D3: (16.9%); D4: (17.2%) CNI + prednisone D3: (8.7%); D4: (9.2%) MMF/Aza + prednisone D3: (0.3%); D4: (0.4%)
Hod et al. [12]	Israel	KT	74	50 (67.6%)	60.2 (53.3–69.8)	3.1 (1.5–8.3)	BNT162b2	ELISA	173 (172–174)	Tac + MPA + prednisone (44.6%) Tac + MPA (14.9%) Tac + prednisone (27%) CyA (5.4%) Aza (4%) mTOR inhibitor (5.4%)
Brandstetter et al. [13]	Austria	KT	Total: 324 D3:147 D4:41	217 (67%)	60.6 (51.4–68.2)	7.0 (3.4–11.3)	BNT162b2 mRNA-1273	ELISA	NA	Tac (77.5%) MPA (70.4%) CyA (10.8%) Aza (8.0%) CS (68.5%) mTOR inhibitor (9.3%)
Osmanodja et al. [17]	Germany	KT	D3: 603 D4: 250	D3: 374 (62%) D4: 167 (67%)	D3: 59 (48–68) D4: 61 (51–70)	D3: 8.2 (3.1–13.5) D4: 7.7 (3.0–12.7)	BNT162b2 mRNA-1273 ChAdOx1-S Ad26.COV2.S	ELISA	64 (55–84)	Tac D3: (73.6%); D4: (73.6%) CyA D3: (16.8%); D4: (16.4%) Belatacept D3: (7.6%); D4: (10%) MPA D3: (93.7%); D4: (50.4%) mTOR inhibitor D3: (1%) Aza D3: (0.8%)
Harberts et al. [14]	Germany	LIT	D3: 106 D4: 36	D3: 64 (60.4%) D4: 23 (63.9%)	D3: 59 (51.0–68.3) D4: 61 (52.5–67.0)	D3: 8.8 (2.6–14.8) D4: 10 (2.6–21.3)	NA	ELISA	126 (93–148)	Tac D3: (17.9%); D4: (16.7%) CyA D3: (1.9%); D4: (2.8%) mTOR inhibitor D3: (21.7%); D4: (11.1%) MMF D3: (43.4%); D4: (44.4%) CNI D3: (65.1%); D4: (63.9%) Aza D3: (1.9%); D4: (2.8%) prednisone D3: (10.4%); D4: (19.4%) ≥3 immunosuppressants D3: (7.5%); D4: (8.3%)

IQR, interquartile range; KT, kidney transplant; LIT, liver transplant; HT, heart transplant; LUT, lung transplant; PT, pancreas transplant; D3, the third dose of COVID-19 vaccine; D4, the fourth dose of COVID-19 vaccine; CLIA, chemiluminescence analysis; ELISA, enzyme-linked immunosorbent assay; NA, not available; Tac, tacrolimus; MPA, mycophenolate; CS, corticosteroids; CyA, cyclosporin A; Aza, azathioprine.

**Table 3:** Efficacy and safety of the third and fourth dose of COVID-19 vaccine among SOT recipients. SOT, solid organ transplant.

Study	Days from D3 to Antibody Measurement	Seropositive Rate of D3 (%)	Seroconversion Rate of D3 (%)	Antibody Titers of D3	Days from D4 to Antibody Measurement	Seropositive Rate of D4 (%)	Seroconversion Rate of D4 (%)	Antibody Titers of D4	Adverse Effect
Karaba et al. [18]	14 d	56%	NA	Anti-RBD IgG 43 (83, 115) BAU/mL Anti-S IgG 42 (5134) BAU/mL	29 (17–38) d	84%	64%	Anti-RBD IgG 255 (97, 873) BAU/mL Anti-S IgG 229 (115, 656) BAU/mL	NA
Peled et al. [19]	173 ± 4 d	61%	NA	Anti-RBD IgG 12.5 AU/mL	16 ± 4 d	81%	47%	Anti-RBD IgG 97 AU/mL	NA
Perrier et al. [16]	122 (72–160) d	68%	22%	NA	32 (28–54) d	76%	47%	NA	NA
Schrezenmeier et al. [20]	NA	NA	NA	NA	32 (28–35) d		76%	NA	NA
Thomson et al. [15]	NA	76%	49%	Anti-S IgG 295 (91,611) BAU/mL	NA	81%	25%	Anti-S IgG 437 (26, 2211) BAU/mL	NA
Hod et al. [12]	3 wk	78%	NA	Anti-RBD IgG 38 (95%CI 21, 71) NAb 66 (95%CI 41, 108)	21 (21, 21) d	95%	75%	Anti-RBD IgG 647 (95% CI 361, 1159) NAb 950 (95% CI 550, 1641)	D4: AEs 82.4% (local AEs 75.7% and systemic AEs 37.8%) Injection site pain 76% Fatigue 27% No episodes of rejection were observed. No allergic responses were documented.
Brandstetter et al. [13]	37 (32–40) d	81%	64%	Anti-RBD IgG 243 (40, 821) BAU/mL	26 (26, 27) d	84%	29%	Anti-RBD IgG 45 (18, 112) BAU/mL	D3: 1 Biopsy-proven acute rejection
Osmanodja et al. [17]	NA	NA	29%	NA	NA	NA	56%	NA	NA
Harberts et al. [14]	29.5 (23–49) d	92%	76%	Anti-RBD IgG 1891 AU/mL	NA	94%	60%	Anti-RBD IgG 1196 AU/mL	D3: pain/swelling, fatigue, headache, vomiting, muscle pain, joint pain, and diarrhea; D4: pain/swelling, fatigue, headache, and muscle pain.

D3, the third dose of COVID-19 vaccine; D4, the fourth dose of COVID-19 vaccine; NA, not available; BAU, binding antibody unit; IgG, immunoglobulin G; RBD, receptor-binding domain antibody; S, spike; AEs, adverse effects; NP, nucleocapsid protein; NAb, neutralizing antibody.

**Table 4:** Clinical characteristics associated with the seroconversion following third and fourth doses of vaccinations.

Study	Factors	Third Dose			Fourth Dose		
		N	Multivariate OR (95% CI)	*p* Value	N	Multivariate OR (95% CI)	*p* Value
Thomson et al. [15]	CNI monotherapy	273	4.48 (2.69–7.63)	<0.0001	97	2.44 (1.11–5.80)	0.033
	Diabetes	200	0.49 (0.32–0.75)	0.001	NA	NA	NA
	Vaccine within the 1st year post-transplant	534	0.28 (0.15–0.54)	0.0001	17	0.57 (0.19–1.87)	0.33
Osmanodja et al. [17]	Low positive anti-S IgG before vaccination	NA	28.5 (7.18–201)	<0.001	NA	18.7 (4.68–134)	0.001
	Age	NA	0.98 (0.96–0.99)	0.016	NA	0.96 (0.94–0.99)	0.004
	BMI	NA	1.06 (1.01–1.11)	0.012	NA	NA	NA
	Transplant age	NA	1.06 (1.03–1.09)	<0.001	NA	1.09 (1.04–1.16)	0.002
	Belatacept	NA	0.15 (0.03–0.45)	0.008	NA	0.03 (0.004–0.13)	<0.001
	MPA dose in MMF equivalent in g	NA	0.29 (0.20–0.43)	<0.001	NA	0.34 (0.18–0.59)	0.001
	eGFR in mL/min/1.73 m^2^	NA	1.02 (1.01–1.04)	<0.001	NA	NA	NA
	Hemoglobin	NA	1.29 (1.13–1.49)	0.001	NA	NA	NA

CNI, calcineurin inhibitor; N, number; OR, odds ratio; NA, not available; BMI, body mass index; MPA, mycophenolic acid; MMF, mycophenolate mofetil; eGFR, estimated glomerular filtration rate.

## Data Availability

The data that support the findings of this study are available from the corresponding author upon reasonable request.
